# Interacting with tumor cells weakens the intrinsic clockwise chirality of endothelial cells

**DOI:** 10.1063/5.0115827

**Published:** 2022-12-06

**Authors:** Benson Hang, Eman Jassem, Hanan Mohammed, Leo Q. Wan, Jason I. Herschkowitz, Jie Fan

**Affiliations:** 1 Department of Natural Sciences, CASL, University of Michigan-Dearborn, Dearborn, Michigan 48128, USA; 2 Department of Biomedical Engineering, Rensselaer Polytechnic Institute, Troy, New York 12180, USA; 3 Center for Biotechnology and Interdisciplinary Studies, Rensselaer Polytechnic Institute, Troy, New York 12180, USA; 4 Department of Biological Sciences, Rensselaer Polytechnic Institute, Troy, New York 12180, USA; 5 Department of Biomedical Sciences, Cancer Research Center, University at Albany-SUNY, Rensselaer, New York 12144, USA

## Abstract

Endothelial cells (ECs) possess a strong intrinsic clockwise (CW, or rightward) chirality under normal conditions. Enervating this chirality of ECs significantly impairs the function of the endothelial barrier. Malignant tumor cells (TCs) undergo metastasis by playing upon the abnormal leakage of blood vessels. However, the impact of TCs on EC chirality is still poorly understood. Using a transwell model, we co-cultured the human umbilical vein endothelial cells or human lung microvascular endothelial cells and breast epithelial tumor cell lines to simulate the TC–EC interaction. Using a micropatterning method, we assessed the EC chirality changes induced by paracrine signaling of and physical contact with TCs. We found that the intrinsic clockwise chirality of ECs was significantly compromised by the TC's physical contact, while the paracrine signaling (i.e., without physical contact) of TCs causes minimal changes. In addition, ECs neighboring TCs tend to possess a left bias, while ECs spaced apart from TCs are more likely to preserve the intrinsic right bias. Finally, we found the chirality change of ECs could result from physical binding between CD44 and E-selectin, which activates protein kinase C alpha (PKCα) and induces pseudopodial movement of EC toward TC. Our findings together suggest the crucial role of EC–TC physical interaction in EC chirality and that weakening the EC chirality could potentially compromise the overall endothelial integrity which increases the probability of metastatic cancer spread.

## INTRODUCTION

More than 1.9 × 10^6^ new cancer cases and 600 thousand cancer deaths are projected to occur in the United States in 2022.^1^ The major cause of cancer morbidity and mortality is metastasis, a process of several intricate steps including tumor cells (TCs) detaching from the primary tumor, intravasating into and extravasating out of the circulatory system before invading a secondary tissue. Tumor cells achieve intravasation and extravasation by exploiting the abnormal leakage of pathological blood vessels.^2,3^ Although the exact mechanism remains unclear, it has been observed that endothelial functions are altered during the early and late stages of cancer progression.^4^

The endothelial cells (ECs) lining the lumen surface of blood vessels control the passage of molecules and cells in and out of the bloodstream, and they, hence, regulate the environment in biological tissues. Normally, the EC layer serves as a barrier with adherens junctions and tight junctions between cells that regulate the permeability of blood vessels. However, the barrier functions can be disrupted remarkably in pathological processes.^5,6^ In metastasis, breakdown of the endothelial barrier is critical for the transmigration of TCs during intravasation and extravasation. It is attributable to both the paracrine^7–12^ and physical receptor-ligand communications between the tumor and ECs,^13–16^ which consequently triggers a cascade of intracellular signals in ECs, not only affecting their growth but also altering their cytoskeletal proteins and promoting asymmetric cell morphology and migration.^7,17^

The handedness of the cell, termed cell chirality, has been recently discovered as an intrinsic property of the cell.^18^ In other words, the cell is left–right asymmetrically structured and functions in a similar way of a human body, but on a much smaller scale. Cell chirality has been found phenotype-specific and seen as left–right biased cell alignment,^18,19^ rotation,^20–23^ migration,^24–26^ and organelle positioning.^27–29^ Cell chirality can notably influence the planar cellular organization in monolayers and account for the organ-specific asymmetries such as the heart,^29^ gut,^27^ and genitalia.^30^ In blood vessels, ECs have been found possessing a strong clockwise (CW, or rightward) chirality,^18,31^ which may lead to a helically aligned tubular sheet. Enervating the rightward chirality of ECs through protein kinase C alpha (PKCα) activation impairs the endothelial barrier function by changing the cell shape, alignment, and cell junctional morphology.^31^ Activation of PKCα in ECs has been found in various vascular conditions, including tumor adhesion.^32^ However, it is unclear of the alteration of the CW chirality of ECs when interacting with TCs during the metastasis.

Using a breast cancer model, we focus on studying the changes in EC chirality caused by paracrine signaling and physical contact of TCs. Any changes or disruptions of intrinsic CW chirality of EC into non-chiral (NC) or counterclockwise (CCW) chirality may compromise the overall endothelial integrity which potentially increases the risk of metastasis. We hypothesized that either physical contact or paracrine signaling between TCs and ECs disrupts the chirality of the endothelial barrier. We hope to provide insight into how TC–EC interaction modulated endothelial morphogenesis may affect trans-endothelial migration during extravasation and aid the identification of therapeutic strategies to stop metastasis.

## RESULTS

### The intrinsic clockwise chirality of endothelial cells is disrupted in TC–EC co-culture

**FIG. 1.**
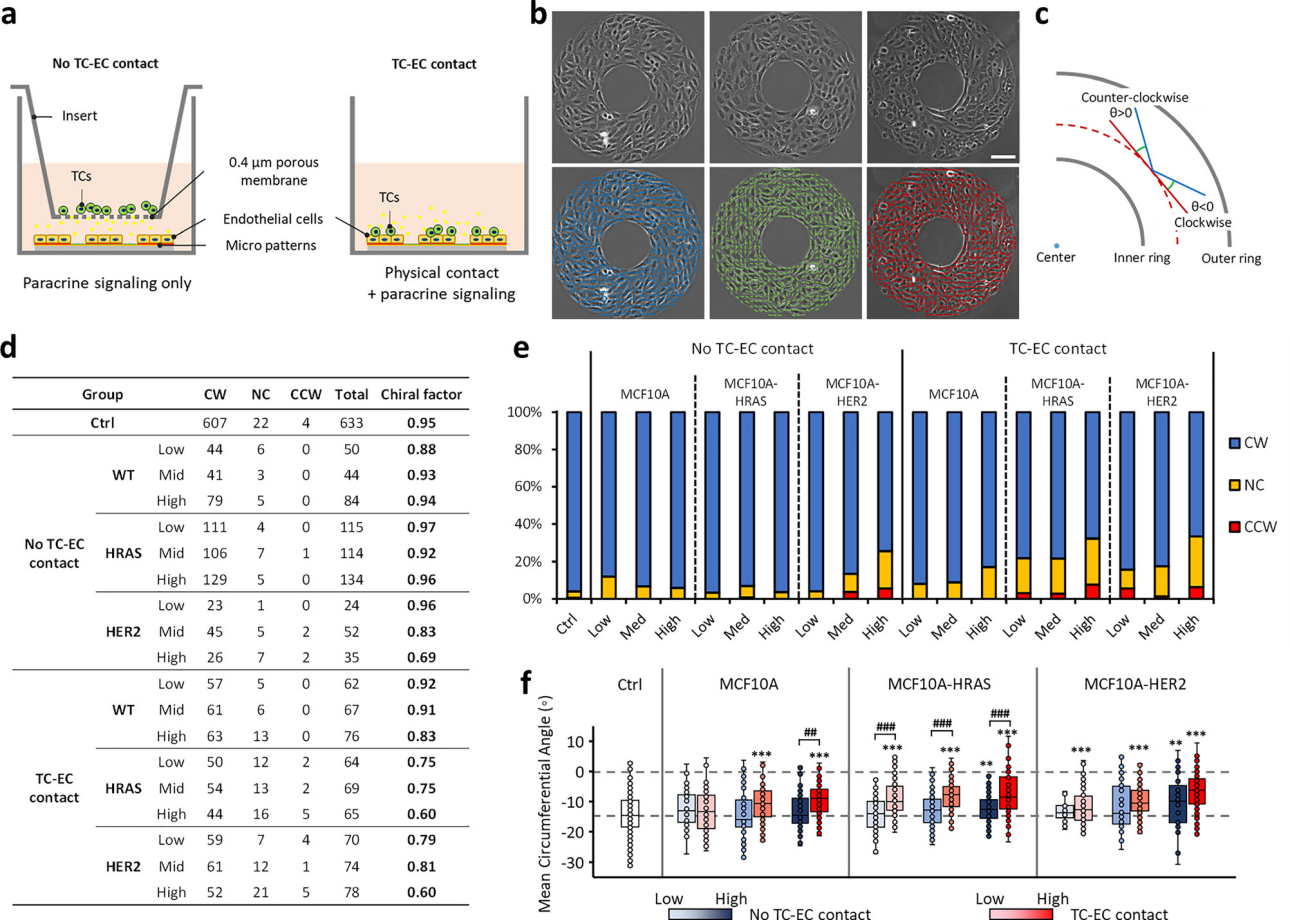
(a) Models of co-cultured ECs and TCs used to study the chirality change of ECs caused by TC paracrine signaling and physical contact during extravasation. (b) Top row: typical images of clockwise (CW), non-chiral (NC) micropatterns formed by ECs under the control condition, or counterclockwise (CCW) micropattern under the physical contact of HRAS TCs; bottom row: the color bars, generated by a pre-developed MATLAB program, show the aligning direction of each cell on the micropattern. (Scale bar: 100 *μ*m). (c) The definition of circumferential angle of each bar [blue bar, cell alignment in (b)] deviated from the circumferential direction (red line) and its relationship to chirality. (d) Numbers of the CW, CCW, and NC rings analyzed in each group and the corresponding chiral factors of ECs treated by paracrine signaling or physical contact of TCs. Chiral factor: defined by comparing ring numbers from the formula: (number of CW rings − number of CCW rings)/number of total rings, with CF = +1 standing for complete CW and CF = −1 for complete CCW. (e) Percentage of CW, NC, and CCW rings of ECs from table (d). (f) Mean circumferential angle of ECs on the ring-shaped micropatterns [illustrated in (c)]. The “low,” “mid,” and “high” in figures. (d)–(f) represent the TC densities (20, 100, and 200k/ml) introduced to ECs in the different co-culture groups. Data in (f) are represented as mean ± SD. Under the control condition, 633 rings of ECs were analyzed; under the non-contact condition of MCF10A cells, 50 (low), 44 (mid), and 84 (high) rings were analyzed; under the contact condition of MCF10A cells, 62 (low), 67 (mid), and 76 (high) rings were analyzed; under the non-contact condition of HRAS cells, 115 (low), 114 (mid), and 134 (high) rings were analyzed; under contact condition of HRAS cells, 64 (low), 69 (mid), and 65 (high) rings were analyzed; under the non-contact condition of HER2 cells, 24 (low), 52 (mid), and 35 (high) rings were analyzed; under contact condition of HER2 cells, 70 (low), 74 (mid), and 78 (high) rings were analyzed. “^*^” in (f) indicates significant differences between the experimental group and control; “^*^” or “#” indicates P < 0.05; “^**^” or “##” indicates P <0.01; and “^***^” or “###” indicates P <0.001 by one-way analyses of variance (ANOVAs) with the Tukey method between groups.

Oncogenic activation of GTPase HRas (HRAS) and human epidermal growth factor receptor 2 (HER2) has been frequently found in breast cancers, leading to tumor initiation, progression, and metastasis [Fig. S1(a)].^33–35^ In this study, the MCF10A human breast epithelial cell line and its malignant mutants, MCF10A-HRAS (or MCF10AT1, HRAS overexpression) and MCF10A-HER2 (HER2 overexpression) [Fig. S1(b)], were co-cultured with ECs in a transwell system to simulate the non-contact or contact TC–EC interactions during the metastasis [Fig. 1(a)]. In addition, each type of TCs was used at a high, mid, or low concentration to simulate different degrees of tumorous conditions. The ECs in co-culture were seeded on a ring-shaped micropattern, and their chirality, including the numbers and percentages of CW, CCW, NC rings [Figs. 1(b)–1(e) and S2], the chiral factor [Fig. 1(d)], and the mean circumferential angle of cell alignment on the micropattern [Fig. 1(f)], were calculated using a custom-written MATLAB (MathWorks) program as described previously.^18^

Normally, human umbilical vein endothelial cells (hUVECs) are strongly CW dominant with a chiral factor above 0.90 [Figs. 1(d) and 1(e)], consistent with previous reports.^31^ Non-contacting co-culture with wildtype MCF10A and HRAS cells did not change the strong CW chirality of ECs, while increasing concentration of HER2 cells induces a 27% decrease in CW chirality of EC shown as the chiral factor decreasing from 0.95 to 0.69 [Figs. 1(d) and 1(e)]. For TC–EC contact groups, the ECs formed notable NC and CCW rings when co-cultured with TCs. In addition, their chiral factor is decreased to 0.83 when co-cultured with MCF10A, and down to a 0.60 level with HRAS or HER2 cells [Figs. 1(d) and 1(e)]. The results from the mean circumferential angle give details about the chiral alignment of cells on the micropattern [Fig. 1(f)]. In both TC–EC non-contacting groups and contacting groups, it shows an overall similar decreasing trend along with the increase in TC density. [Note that the circumferential angle for CW cell alignment is negative in Fig. 1(f).] Under non-contacting co-culture, it requires a high TC concentration to induce a significant decrease in the chiral alignment of ECs; however, under TC–EC contacted co-culture, low density of HRAS or HER2 overexpression cells was adequate to make differences [Fig. 1(f)], but not the wildtype. Particularly for HRAS cells, the physical contact caused a significantly stronger interruption of the CW chirality of ECs than paracrine signaling.

Lung is one of the most common target organs for metastasis of malignant breast cancer.^36^ Therefore, we also examined the effects of breast cancer cells on the chirality of the human lung microvascular endothelial cells (hLMVECs). Under the normal condition, the hLMVECs exhibit a strong CW chirality with a chiral factor of 0.88 [Fig. S3(a)], comparable to 0.95 of the hUVECs. Co-cultured with different TCs, the hLMVECs exhibit quite similar patterns of CW chirality weakening [Figs. S3(a) and S3(b)]. Particularly under the physical contact of the HRAS and HER2 cells, the chiral factors of hLMVECs dropped to 0.66 and 0.50 [Fig. S3(a)], along with significant decreases in the mean circumferential angles [Fig. S3(c)], again consistent with the observations from hUVECs. These together suggest the TCs could cause the weakness of the CW chirality of ECs by interacting with them under a physical contact condition. This effect could be significantly enhanced by the oncogenic expression of HRAS and HER2, which may contribute to their malignancy in metastasis.

### Tumor secretion plays a limited role in endothelial cell chirality

**FIG. 2.**
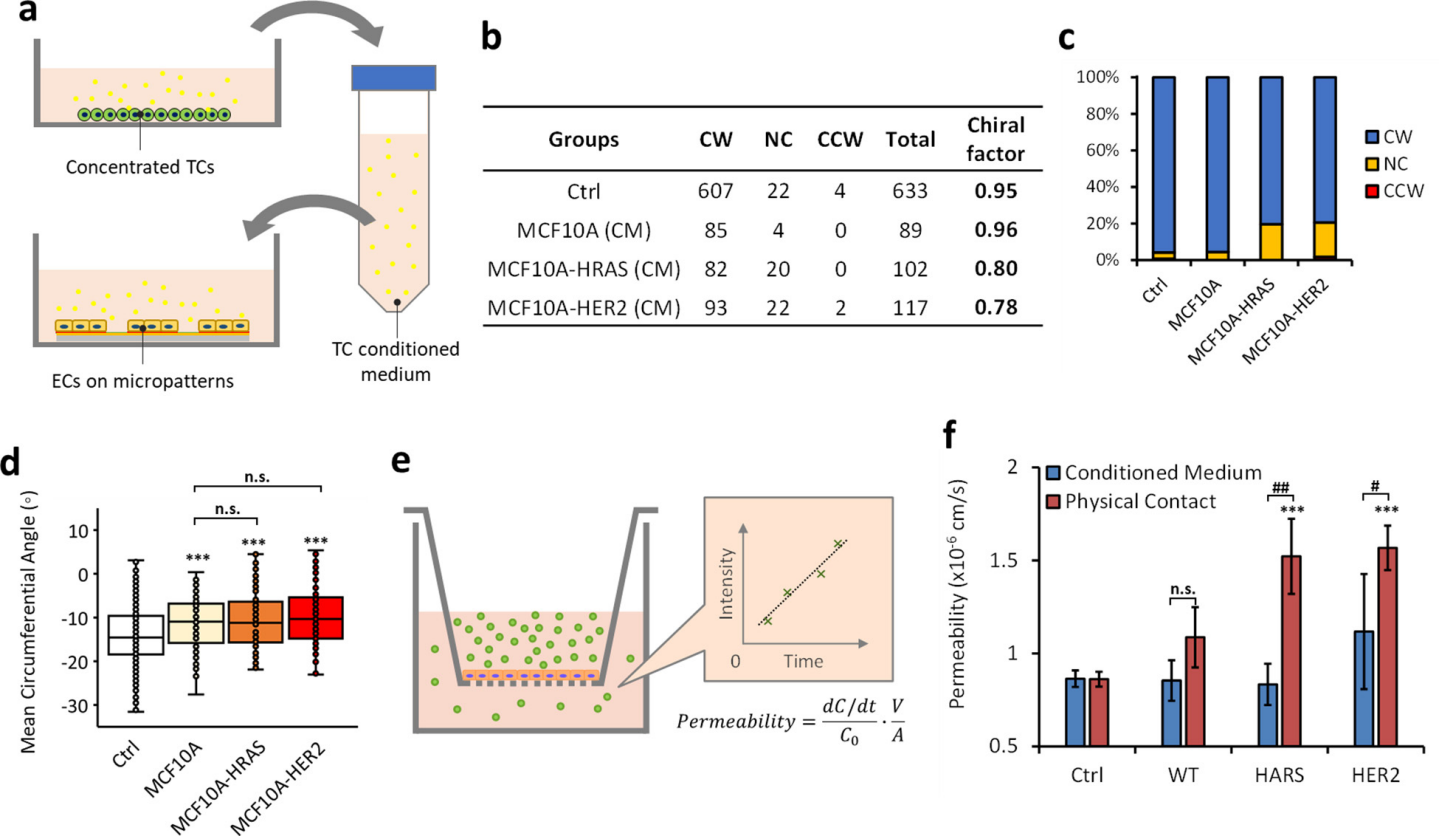
(a) Schematic representation of testing the chirality of ECs treated by TC conditioned medium. (b) Numbers of the CW, CCW, and NC rings and the corresponding chiral factors of ECs treated by TC conditioned medium. “CM” is an abbreviation for “conditioned medium” in group names. (c) Percentage of CW, NC, and CCW rings of ECs from table (b), showing the increased non-chiral chirality of ECs treated with HRAS or HER2 TC conditioned medium. (d) Mean circumferential angle of ECs on the ring-shaped micropatterns, which, however, does not show significant differences. Data are represented as mean ± SD. Under normal medium, 633 rings of ECs were analyzed; under MCF10A, HRAS, or HER2 cell conditioned medium, mean circumferential angles of ECs from 89, 102, or 117 rings were analyzed, respectively. (e) Schematic diagram of endothelial monolayer permeability measurement using a transwell model. (f) Permeability of the hUVEC monolayer with TC conditioned medium or TC physical contact. Data are presented as mean ± SD (n = 4). “^*^” in (d) and (f) indicates significant differences between the experimental group and control; “^*^” or “#” indicates P <0.05; “^**^” or “##” indicates P <0.01; and “^***^” or “###” indicates P <0.001 by one-way analyses of variance (ANOVAs) with the Tukey method between groups. “n.s.” stands for no significant difference.

In the TC–EC contact condition, the cell interactions are not only from physical bindings but also from the paracrine signaling of TCs simultaneously. In addition, the TC–EC communications are bi-directional even without the physical contact of the two cell types.^9^ It means TC secretions are modifying ECs, while EC secretions are simultaneously stimulating TCs in a cycle. Therefore, we further investigated the EC chirality changes in the TC conditioned medium (CM) to understand whether the weakness of EC chirality results from inherent tumor secretions which do not require the EC induction [Fig. 2(a)]. When cultured in TC conditioned medium, the ECs formed an increased percentage of NC rings, and their chiral cell alignment was significantly decreased compared with control [Figs. 2(b)–2(d)]. However, the decreases of either overall EC chiral factors or the chiral cell alignment caused by the inherent tumor secretions are not comparable with the physical contact groups in Figs. 1(d)–1(f). In addition, neither HRAS nor HER2 conditioned medium induces significantly weaker CW chiral alignment of ECs compared with the wildtype [Fig. 2(d)]. Considering the highest density of TCs was used to generate this conditioned medium, therefore, the inherent secretions of TCs play a limited role in EC chirality change under the TC–EC physical contact condition.

The cell chirality is closely associated with the integrity of the EC monolayer. Its permeability increases with the weakening of CW chirality and peaks when ECs become completely non-chiral.^31^ The results from a permeability assay show that the TC conditioned medium is not sufficient to induce a significant change in EC permeability [Figs. 2(e) and 2(f)], suggesting the limited influence of TC secretions on the EC chirality. On the other hand, in the physical TC–EC co-culture groups where EC chirality was weakened, significant increases in EC permeability were also observed, as expected [Fig. 2(f)].

### Physical contact with tumor cells disrupts the chiral alignments of endothelial cells

**FIG. 3.**
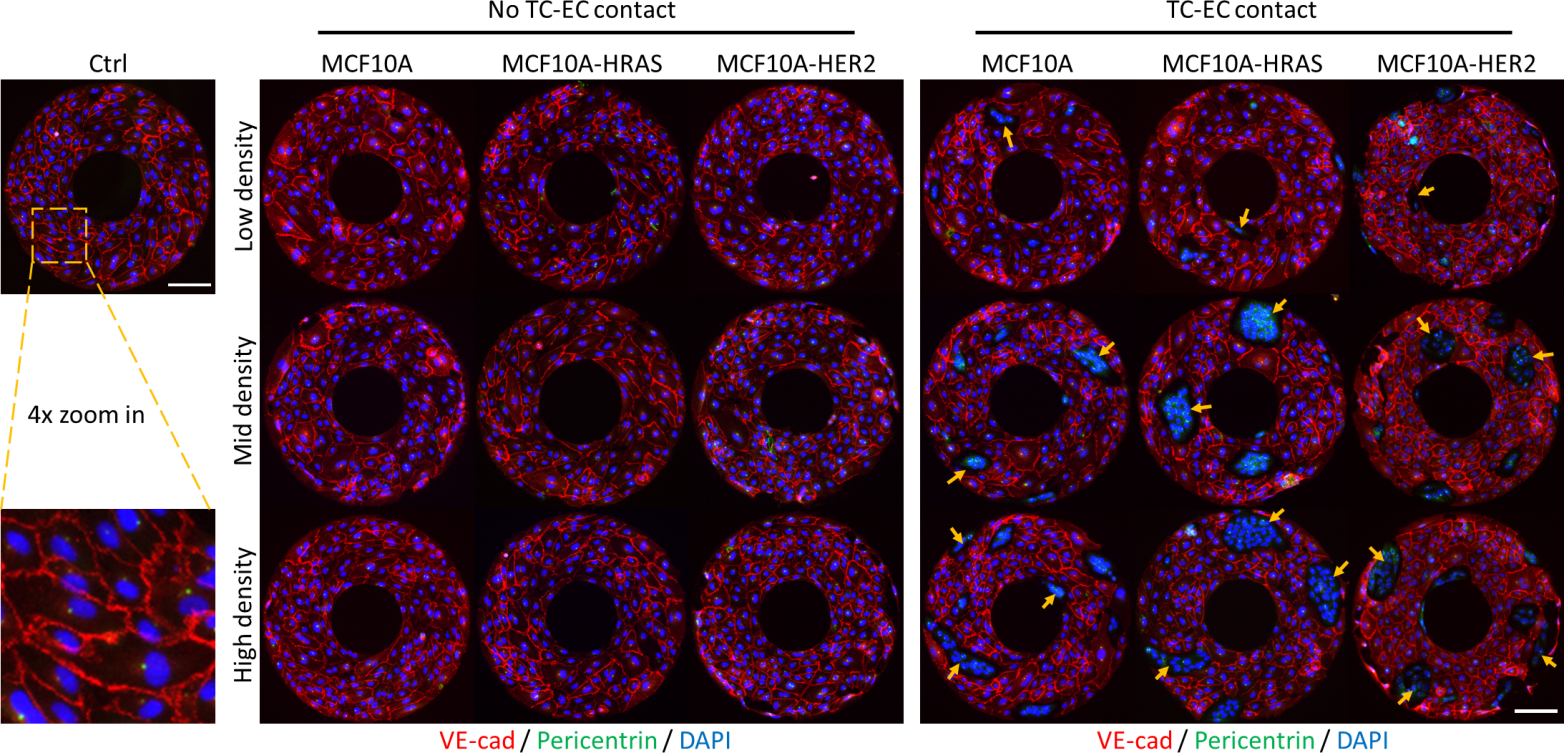
Immunofluorescence images of micropattern ECs with TC paracrine signaling (left) or physical contact (right). Staining shows the EC nuclei (DAPI, blue), centrosomes (AF488 pericentrin, green), and adherens junctions (AF594 VE-cad, red). The physical contact with TCs (MCF10A-HRAS, MCF10A-HER2) disrupts the CW dominant EC chirality. The tumor cells are indicated by yellow arrows. Scale bar in (a): 100 *μ*m.

The integrity of cell–cell junctions plays an important role in the collective migration of planar cells to form multicellular chiral alignment on the micropattern.^25^ Previously, we have found significant local misalignment of ECs occurred on the ring-shaped micropattern when they became non-chiral.^31^ In this study, it is shown in fluorescent images that the ECs form a well-arranged monolayer on the micropatterns with intact cell–cell junctions between adjacent cells under the control condition (Fig. 3). With only tumor secretion in TC–EC non-contact groups, the ECs kept relatively intact and continuous cell–cell junctions, while the endothelial monolayer is significantly interrupted with physical involvement of TCs, and misalignment of ECs occurs at the boundary of TCs, suggesting the ECs with direct contact with TCs may possess inconsistent cell chirality.

### Physical contact of tumor cells compromises the left–right biases of endothelial cells

**FIG. 4.**
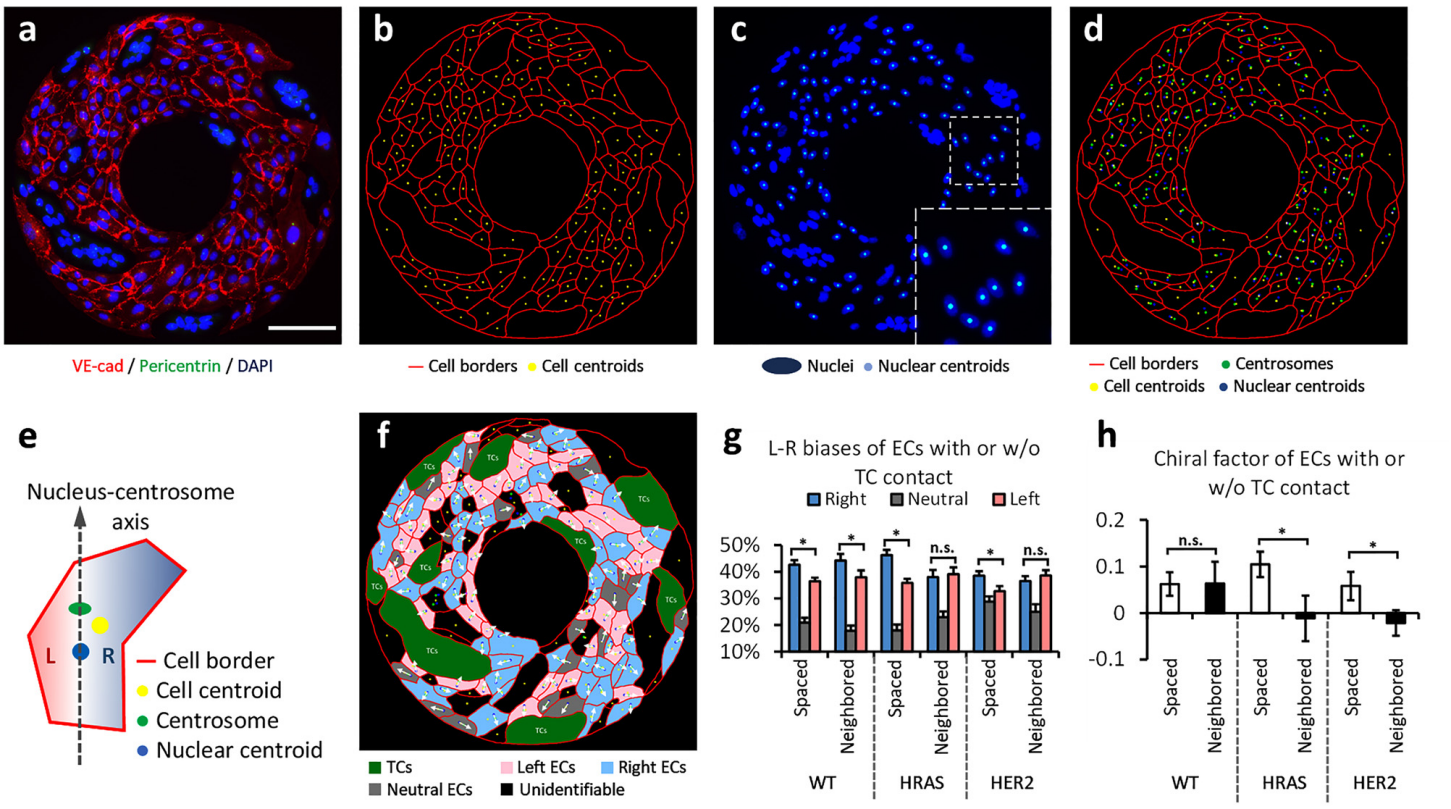
(a) Immunofluorescence of ECs on a ring-shaped micropattern showing cell adherens junctions (VE-cad, red), cell nuclei (DAPI, blue), and centrosomes (pericentrin, green). (b) Cell borders segmented from the red channel in (a), shown with the calculated cell centroids (yellow). (c) Cell nuclei (blue) segmented from the blue channel in (a), shown with nuclear centroids (cyan). (d) Merged image for cell bias analysis, including cell borders (red), centrosomes (green), nuclear centroids (blue), and cell centroids (yellow). (e) A schematic diagram of determination of the left (L) or right (R) cell bias according to the positioning of the cell centroid relative to the nucleus-centrosome vector. (f) Color-coded cells by their biases on the micropattern. (g) and (h) L–R biases and the corresponding chiral factors of ECs with or without direct TC contact. Data are represented as mean ± SE. In the MCF10A-WT group, 16 rings and 1222 TC-spaced ECs were analyzed; 16 rings and 382 TC-neighbored ECs were analyzed; in the MCF10A-HRAS group, 15 rings and 1094 TC-spaced ECs were analyzed; 15 rings and 327 TC-neighbored ECs were analyzed; in MCF10A-HER2 group, 15 rings and 1417 TC-spaced ECs were analyzed; 15 rings and 325 TC-neighbored ECs were analyzed. “^*^” indicates P <0.05; “^**^” indicates P <0.01; and “^***^” indicates P <0.001 by student t-test. “n.s.” stands for no significant difference. Scale bar in (a): 100 *μ*m.

The individual cell chirality can be reflected by the biased positioning of cell organelles.^24,27,29,31^ We have demonstrated CW rings contain mainly right-biased cells, while CCW rings contain significantly more left-biased cells.^28^ Each fluorescent image of micropatterned EC monolayer was further segmented into cell borders, cell centroids, nuclear centroids, and centrosomes as described in Methods section [Figs. 4(a)–4(d)]. The left–right biases of individual ECs were then determined by the positional bias of the cell centroid relative to the nucleus-centrosome axis to study the role of TC physical contact in EC chirality [Figs. 4(e) and 4(f)]. The ECs spaced from TCs (without direct contact with TCs) show a significant right bias and a positive chiral factor which is consistent with their CW chirality [Figs. 4(g) and 4(h)]. A neighboring relationship with MCF10A cells did not affect the right bias of ECs; however, those ECs neighbored with HRAS or HER2 cells are lack of left–right bias, and their chiral factors change into negative values close to zero, indicating a slight reversal of the intrinsic chiral bias of ECs [Figs. 4(g) and 4(h)]. These results together suggest the chirality of ECs is significantly altered by the physical contact of malignant TCs with HRAS and HER2 overexpression.

### TC–EC physical contact promotes the binding of CD44 and E-selectin, activates PKCα, and induces pseudopodial movement of EC toward TC

**FIG. 5.**
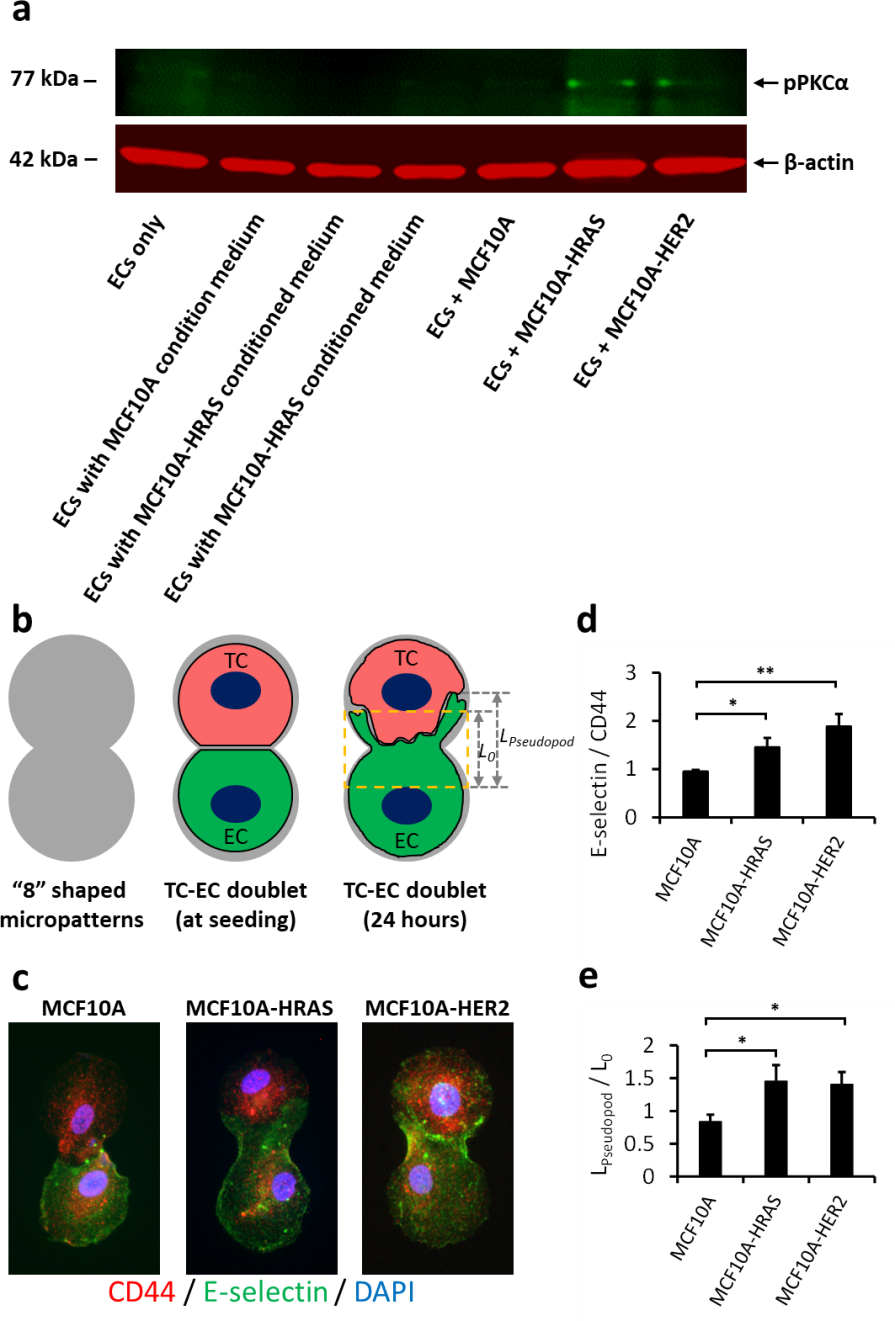
(a) Western blots showing the phosphorylated PKCα of endothelial cells treaded with TC conditioned medium or physical contact. (b) An “eight”-shaped micropattern used to seed single TC–EC doublet and study their physical interactions. (c) Immunofluorescence of TC–EC doublets on the “eight”-shaped micropattern showing TC surface receptor (CD44, red), EC surface adhesion molecule (E-selectin ligands, green), and cell nuclei (DAPI, blue). (d) Colocalization of CD44 and E-selectin calculated in the area between two cells [shown as the yellow-dashed box in (b)]. (e) The extension of EC pseudopods toward TC calculated as L_pseudopod_/L_0_ shown in (b). Data are represented as mean ± SE. 18 pairs of MCF10A-EC, 18 pairs of HRAS-EC, and 12 pairs of HER2-EC doublets were analyzed in (d) and (e). “^*^” indicates P <0.05; “^**^” indicates P <0.01; and “^***^” indicates P <0.001 by one-way analyses of variance (ANOVAs) with the Tukey method between groups. Scale bar in (c): 20 *μ*m.

CD44 is a multifunctional cell surface adhesion receptor that is commonly expressed in MCF10A cell series.^37–40^ It has been reported that CD44 binding to its EC surface ligands, E-selectin, activates the PKCα signaling in ECs.^32^ We have demonstrated previously that PKCα signaling is involved in EC chirality regulation.^31^ In this study, we found a significant elevation of phosphorylated PKCα in ECs when co-cultured with HRAS or HER2 overexpressed TCs rather than the wild type, but the PKCα was inactivated by TC secretions, suggested by undetectable pPKCα bands in any groups of the TC conditioned medium [Fig. 5(a)]. Thus, PKCα activation in ECs requires the physical contact of TCs during the interactions.

In most of the co-cultured models, each cell was physically communicating with multiple neighboring cells; therefore, the left–right bias is an integrated result of the physical stimulations from all surrounding cells. To further study the physical TC–EC interaction within one pair of TC and EC without the interference of other cells, we generated a cell doublet model by patterning single TCs and ECs on the “eight”-shaped micropatterns [Fig. 5(b)]. Interestingly, we found the EC moved its pseudopodia toward TC and formed E-selectin-CD44 binding, rather than the TC invading into the EC [Figs. 5(b) and 5(c)]. The malignant HRAS and HER2 oncogenes increased the physical interaction between the TC and EC, shown by the significantly higher colocalization of E-selectin/CD44 in these groups [Fig. 5(d)]. Furthermore, compared with the MCF10A-WT, the HRAS and HER2 overexpressed TCs induced significant increases in EC pseudopodial movement toward the TC [Fig. 5(e)], which could potentially interfere with the chiral alignment of ECs in physical TC–EC interaction.

## DISCUSSION

During metastasis, close interaction between TCs and ECs occurs during the intravasation and extravasation, eliciting the endothelial barrier remodeling to enable the transmigration of metastatic TCs.^41^ In tumor blood vessels, the endothelial barrier is modulated by tumor secretions, but their role in cell chirality is limited. It is consistent with our previous reports that common growth factors or inflammatory factors do not change the EC chirality since ECs are constantly exposed to various cytokines in the bloodstream. However, it is also possible that accumulated secretions from HER2-positive tumors at the local microenvironment of interstitial space or micro blood circulation may induce non-chiral ECs on the blood vessels. Compared with paracrine signaling, the physical contact between the EC and TC could significantly weaken the intrinsic CW chirality of ECs by binding the tumor surface adhesion molecules to their ligands on the EC surface.

The non-contacting TC–EC co-culture results in more complicated communications between the two cell types, than the monoculture of ECs with the TC conditioned medium. In the case of non-contacting co-culture, the communication between TCs and ECs is bi-directional and continuous during the period when the TCs have paracrine effects on ECs at the same time when the ECs exert paracrine effects on TCs. It allows both cell types to alter their paracrine signaling and reactions constantly to the stimuli along with culture time. In the case of monoculture of ECs with the TC conditioned medium, the communication is unidirectional and only one-shot with the inherent tumor secretions from TCs to ECs without potential adjustments over time. This phenomenon can be reflected by the results from Figs. 1(d) and 2(b) that the TCs with HER2 overexpression results in a decrease in CW chirality of ECs under the non-contact co-culture condition, but not under any TC conditioned medium, which suggests the importance of bi-directionality and continuity in TC–EC communication in this process.^8,42,43^ Compared to HER2, a transmembrane glycoprotein receptor sensing various paracrine signals, the HRAS is a GTPase enzyme completely enclosed in the TC cytoplasm. The HER2 overexpression in TCs may, therefore, involve more active communication with ECs and induce more significant alteration in chirality.

PKC signaling pathway is known as the chirality regulatory mechanism in ECs. Activation of PKC at an intermediate level changed the EC chirality from CW to NC, resulting in the endothelial leakage; while hyperactivation of PKC may further induce a CCW chirality which may re-align the cells in a reversed pattern and re-enforce the endothelial barrier.^31^ In this study, we found physical contact with TCs is responsible for the activation of PKCα of ECs, not the TC conditioned medium, confirming that the chirality changes of ECs were due to physical contact with TCs. Furthermore, only weakening of CW chirality was observed, suggesting the tumor-induced PKC activation was within the range of weakening the CW chirality, not re-establishing the CCW chirality of ECs. Therefore, this weakening in CW chirality of ECs only leads to the disruption of the uniformity of the endothelial alignment on the lumen of blood vessels, which may potentially increase the nutrient delivery in tumors and the risk of TC transmigration into the circulation system. Moreover, loss of chirality may induce misaligned collective migration of ECs with their neighbors,^17^ which may potentially enhance the blood vessel branching during tumor-induced angiogenesis.

It has been found recently when ECs completely lost their CW chirality and became non-chiral, their orderly multicellular alignment was significantly interrupted, with openings and overlaps between adjacent cells,^31^ a similar endothelial morphology as reported in tumor blood vessels.^2^ It is probable that the resultant misalignment of ECs can promote TC transmigration, despite the fact that transendothelial migration also relies on the malignancy of TCs. Both HRAS and HER2 oncogenes enhance the tumor-induced EC chirality weakening compared with the MCF10A wild type. This could be a result of their amplified invasive activity and motility^44–47^ which promotes the dynamic physical interactions with ECs, but the roles of HRAS and HER2 may be different. Specifically, the paracrine signaling of HER2 cells could impact the EC chirality, while HRAS cells did not. It may be because the HER2 increases the cancer autocrine secretion.^48^ However, the increased autocrine secretion is not unidirectional from the HER2 TCs to ECs. Instead, paracrine signaling of ECs must also play a role in activating HER2 on TCs. The underlying mechanism is worth further investigation.

In summary, the endothelium has important implications in metastasis. The strong intrinsic CW chirality of EC acts as a supporter of the integrity of the barrier; however, it could be significantly weakened during physical interaction with TCs under the circumstance of tumor invasion or extravasation. PKCα activation induced by binding of CD44 and its ligands E-selectin on the endothelial surface leads to disruption of EC chirality. In addition, the TC–EC interaction induces pseudopodial movement of ECs toward TC, which may also contribute to the chirality disruption. Such mechanisms constitute a crucial strategy for the development of effective anti-metastatic therapeutics by preserving the intrinsic EC chirality by the inhibition of CD44/E-selectin or PKCα.

## METHODS

### Cell culture

Human umbilical vein endothelial cells (hUVECs, Lonza) or human lung microvascular endothelial cells (hLMVECs, Lonza) were cultured in EGM-2 completed media before passage ten. Human breast epithelial tumor cells (MCF10A wildtype and mutants: MCF10A-T1 with HRAS overexpression and MCF10A-HER2 with HER2 overexpression) were cultured in Dulbecco's Modified Eagle Medium/Nutrient Mixture F-12 (DMEM/F12) supplemented with 5% horse serum, 20 ng/ml epidermal growth factor (EGF), 0.5 mg/ml hydrocortisone, 100 ng/ml cholera toxin, 10 *μ*g/ml insulin, and 100 U/ml penicillin–streptomycin. Multiple common media were tested for co-culture of TCs and ECs, including DMEM-high glucose medium (DMEM-HG, supplemented with 10% fetal bovine serum, 100 U/ml penicillin–streptomycin) and medium 199 (M199, supplemented with 10% fetal bovine serum and 100 U/ml penicillin–streptomycin), as well as the EGM-2 and DMEM/F12 medium mentioned above (Fig. S4). The EGM-2 culture medium was used for the co-culture of ECs and TCs to stimulate the situation of TCs invading blood vessels. TC conditioned medium was generated by 200k cells per milliliter of EGM-2 completed medium under monoculture of TCs for 24 h.

### Microcontact printing

Cell adhesive micropatterns were generated using contact printing as described in our previous study.^18,31^ Briefly, a photomask was created with the ring-shaped feature (200 *μ*m inner diameter and 500 *μ*m outer diameter) or eight-shaped feature (two circles of 50 *μ*m in diameter with 10% overlap in diameter) (CAD/Art Services, Bandon, OR). After that, a master mold containing an array of the specific patterns was fabricated with SU-8 photoresist on a silicon wafer through photolithography. The polydimethylsiloxane (PDMS) stamp was then molded by incubating PDMS and the curing solution mixture at a ratio of 10:1 on the wafer. Then, Ti-Au coated glass slides (LGA Thin Films, Santa Clara, CA) were printed with 1-octadecanethiol (MilliporeSigma) using the PDMS stamp; subsequently, the printed slides were treated by the non-adhesive EG3 [HS-(CH2)11-EG3] (ProChimia Surfaces) for at least 3 h followed by a fibronectin (50 *μ*g/ml, MilliporeSigma) coating for 30 min to enhance the cell attachment (Fig. S5).

### Cell seeding and chirality analysis

For cell chirality studies, the EC suspension (200k/ml) was added onto the ring-shaped micropatterns for initial cell attachment. After 15 min, the extra cells were gently washed off from the slides. For TC–EC non-contact co-culture, the specific TCs were added into a transwell insert with 0.4 *μ*m pores (Corning, NY) at different densities: low (20k/ml), mid (100k/ml), or high (200k/ml) on top of the patterned EC samples; for TC–EC contact co-culture, TCs with the same density were directly added to the top of patterned ECs [Fig. 1(a)]. After 15 min, the extra TCs were gently washed off from the slides. Monoculture of micropatterned ECs was performed as a control. When reaching confluency after 24 h, samples were fixed, and high-resolution phase-contrast images were captured using a Nikon Eclipse Ts2 microscope. The chirality analysis was performed using a custom-written MATLAB (MathWorks) program as described previously.^18^ The cell chirality was determined as clockwise (CW, rightward), counterclockwise (CCW, leftward), or non-chiral (NC) based on the circular statistics of the distribution of cell alignment deviating from circumferential direction [Figs. 1(b) and 1(c)]. The histograms of negatively, positively, or neutrally distributed circumferential angles demonstrated the dominance of CW, CCW, or non-biased cell alignment on a ring (Fig. S2). The subregional angles of cell alignment at different radial positions exhibit the stronger biases of cell alignment at the interior area of a ring than at the edges [Figs. S2(c), S2(f), and S2(i)].

For paired TC–EC interaction studies, TCs and ECs were mixed at a 1:1 ratio before seeding onto the eight-shaped micropatterns following a similar protocol as above. Cell doublets were cultured for 24 h before fixation and immunofluorescence.

### Immunofluorescence

Micropatterned cell samples were fixed with 4% paraformaldehyde, permeabilized with 0.2% Triton X-100/phosphate-buffered saline (PBS) for 15 min, blocked with 10% normal goat serum in 0.1% Triton X-100/PBS for 1 h, and incubated with primary antibodies at 4 °C overnight followed by a 1-h incubation with corresponding secondary antibodies at room temperature. Samples requiring cell nuclei staining were mounted in fluoromount-G with 4′,6-diamidino-2-phenylindole (DAPI) (SouthernBiotech) and imaged using a Nikon Eclipse Ts2 fluorescence microscope. Primary antibodies include mouse anti-VE-cadherin (CD144, Invitrogen) for EC junction, rabbit anti-pericentrin (Thermo Fisher Scientific) for cell centrosomes, mouse anti-CD44 (Sino Biology) for TC surface adhesion molecules, and rabbit anti-E-selectin (CD62e, Sino Biology) for endothelial surface ligands.

To verify the confluency of hUVECs used in permeability measurement, the membranes with cells were cut off carefully from the transwell chamber with a blade after cell fixation. Then, the samples were stained with anti-VE-cadherin (CD144, Invitrogen) and anti-ZO-1 (1A12, Invitrogen) followed by corresponding secondary antibodies and then mounted with fluoromount-G with DAPI (SouthernBiotech) for imaging. Fluorescent images show the hUVECs form intact monolayer on transwell membrane before being treated with TCs or conditioned medium for permeability assays (Fig. S6).

### Image analysis for cell biases

For each fluorescent image of micropatterned ECs [Fig. 4(a)], manual segmentation was first performed to separate individual cells following the cell junction staining [Fig. 4(b), red]. Positions of cell centroids [Fig. 4(b), yellow] were calculated using ImageJ (NIH). Similarly, the nuclear centroids [Fig. 4(c), cyan] were calculated using ImageJ based on the DAPI staining [Fig. 4(c), blue]. Subsequently, channels of cell borders (red), cell centroids (yellow), nuclear centroids (blue), and the centrosome (green) were merged into a single image [Fig. 4(d)]. Finally, the left–right bias of each cell in the endothelial layer was determined by the positional bias of the cell centroid relative to the nucleus-centrosome axis. An EC was considered neutral or non-biased when the cell centroid overlapped with the nucleus-centrosome axis [Fig. 4(e)]. Left–right biased or non-biased ECs were color-coded, with TCs labeled in green [Fig. 4(f)].

### Permeability measurement

The permeability measurement was performed as described previously.^11,31,49^ The hUVECs were placed on fibronectin-coated Transwells (12-well, 0.4-*μ*m pore; Corning) at 1200 cells/mm^2^ and cultured for 24 h to reach confluency. For TC–EC physical contact groups, subsequently, 200k/ml TCs (WT, HRAS, or HER2) were added to the monolayer in the upper chamber of the transwell for 8 h before the permeability measurement; for conditioned medium treatment, the TC conditioned medium (mentioned above) was added to both top and bottom chambers of the transwell, and culture for 8 h before the assay. Upon assay starting, the culture medium (or conditioned medium) with 8 *μ*M fluorescein isothiocyanate (FITC)-bovine serum albumin (BSA) (MilliporeSigma) was added to the upper chamber, while only the culture medium (or conditioned medium) was in the lower chamber [Fig. 2(e)]. Every 30 min for a total duration of 120 min, 100 *μ*l of the sample solution was collected from the lower chamber followed by an immediate refill with fresh culture medium (or conditioned medium). The FITC-BSA concentration of collected samples was determined by the plate reader (Tecan Infinite 200 Pro) with excitation/emission wavelengths of 490/525 nm. The permeability was calculated as

$$P_{\mathrm{BSA}}=\frac{\Delta C/\Delta t}{C_{0}}\cdot \frac{V}{A},$$

where Δ*C*/Δ*t* is the rate of the FITC-BSA concentration increase in the lower chamber during the time interval Δ*t*, *C*_0_ is the FITC-BSA concentration in the upper chamber, assumed to be constant during the experiment, *V* is the medium volume in the lower chamber, and *A* is the area of the endothelial monolayer.

### Western blot

Cell samples were cultured in T25 flasks for 24 h and then lysed on ice using lysis buffer (0.125 M tris at pH 6.8, 4% sodium dodecyl sulfate, and 20% glycerol) with protease and phosphatase inhibitor cocktail (MilliporeSigma). Total protein was quantified using a bicinchoninic acid (BCA) protein assay kit (Thermo Fisher Scientific) and a plate reader (Tecan Infinite 200 Pro). Samples with equal amounts of total protein were subjected to 4%–12% Tris-Glycine gel electrophoresis and transferred to polyvinylidene fluoride (PVDF) membranes. Then, the blots were blocked with 5% BSA in tris-buffered saline with Tween 20 and incubated with rabbit anti-HRAS (Thermo Fisher Scientific), mouse anti-HER2 (Thermo Fisher Scientific), rabbit anti-pPKCα (Thr638, Thermo Fisher Scientific), or mouse anti-β-actin (Invitrogen) followed by the corresponding Alexa Fluor 680 or 790 conjugated secondary antibodies. The fluorescent bands were imaged using a fluorescent imager (LI-COR Odyssey FC).

### Statistics

Data were presented as mean ± SE unless indicated otherwise. One-way ANOVA with the Tukey method was performed for multiple comparisons. Significant differences were accepted at P <0.05.

## SUPPLEMENTARY MATERIAL

See the supplementary material for the additional data, including verification of HRAS and HER2 overexpression in MCF10A cells (Fig. S1), distribution of cell alignment angles on the ring-shaped micropatterns and typical phase contrast images of ring-shaped micropatterns (Fig. S2), TC-induced hLMVECs chirality results (Fig. S3), medium test results for TC–EC co-culture (Fig. S4), schematic diagram of micropatterning (Fig. S5), and fluorescent images of confluent hUVEC monolayers on transwell membrane (Fig. S6).

## Data Availability

The data that support the findings of this study are available from the corresponding author upon reasonable request.
